# Supplementary material for: Intercalation-driven ferroelectric-to-ferroelastic conversion in a layered hybrid perovskite crystal
Source: Nat Commun. 2022 Jun 3;13:3104. doi: 10.1038/s41467-022-30822-6 (PMC9166815; doi:10.1038/s41467-022-30822-6)
Supplement: Supplementary file 1 — Supplementary Information [file 41467_2022_30822_MOESM1_ESM.pdf]

Intercalation-driven ferroelectric-to-ferroelastic conversion in a layered hybrid perovskite crystal

Zhenyue Wu[1,4], Shunning Li[2,4], Yasmin Mohamed Yousry[3], Walter P.D. Wong[1], Xinyun Wang[1], Teng Ma[1], Zhefeng Chen[2], Yan Shao[1], Weng Heng Liew[3], Kui Yao[3], Feng Pan[2,*], Kian Ping Loh[1,*]

1 Department of Chemistry, National University of Singapore, Singapore, Singapore.

2 School of Advanced Materials, Peking University Shenzhen Graduate School, 518055 Shenzhen, P.R. China

3 Institute of Materials Research and Engineering, A*STAR (Agency for Science, Technology and Research), 2 Fusionopolis Way, Innovis, Singapore, 138634 Singapore.

Supporting Information

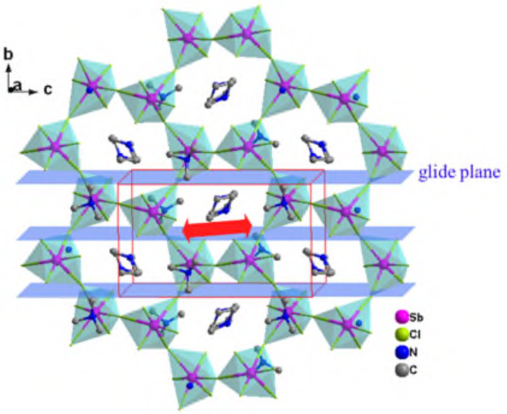

Figure S1. For TSC, the corner-sharing $SbCl_6$ octahedra construct the infinite and honeycomb perovskite layer lying in the *bc*-plane. The red arrow that is located in the glide plane σ_h represents the polarization direction of TSC.

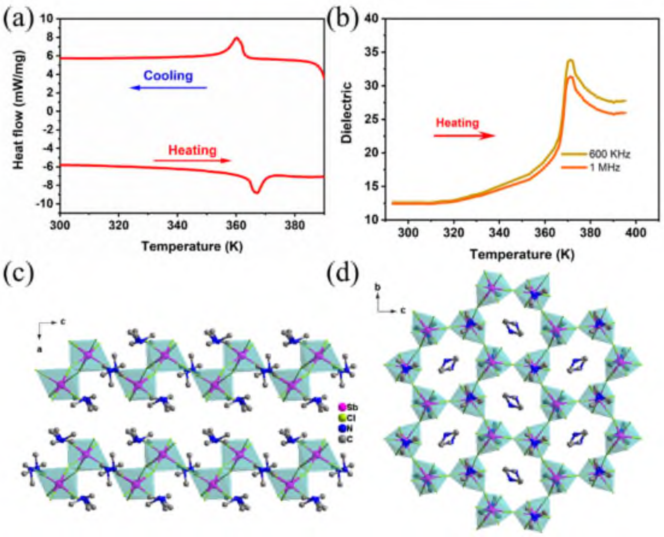

Figure S2. (a) DSC curves for TSC. (b) Variable-temperature dielectric constant of TSC in heating mode. Perspective view of TSC in high temperature paraelectric phase along (c) *b*-axis and (d) *a*-axis.

DSC and variable-temperature dielectric constant reveal that TSC has a structure phase transition at 363 K. In high temperature (380 K) paraelectric phase, TSC crystallizes in $P2_1/c$ space group with point group 2/*m*. These organic TMA cations are highly disordered and have 2-fold axis symmetry. By combining the glide plane and 2-fold axis, the crystallographic inversion center of TSC is thus formed, which is consistent with the centrosymmetric structure. The symmetry breaking of TSC conforms with the Aizu notation 2/*mFm*, indicating that TSC is a uniaxial ferroelectric.

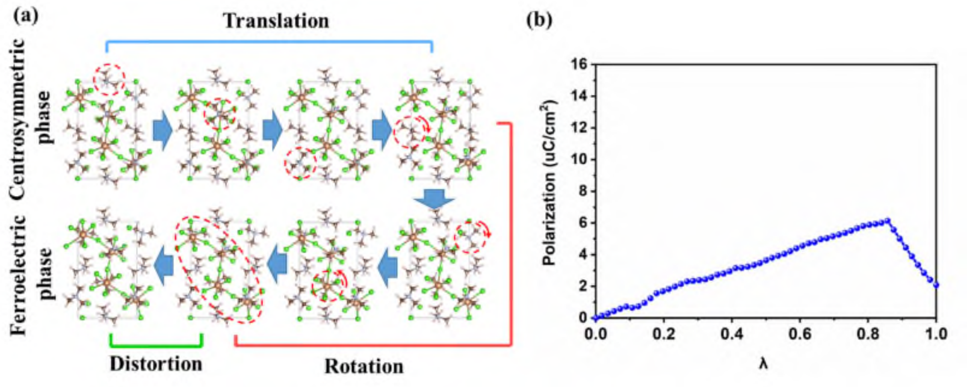

Figure S3. (a) The dynamic path of ferroelectric phase transition for TSC. (b) The polarization value was acquired by Berry phase calculations.

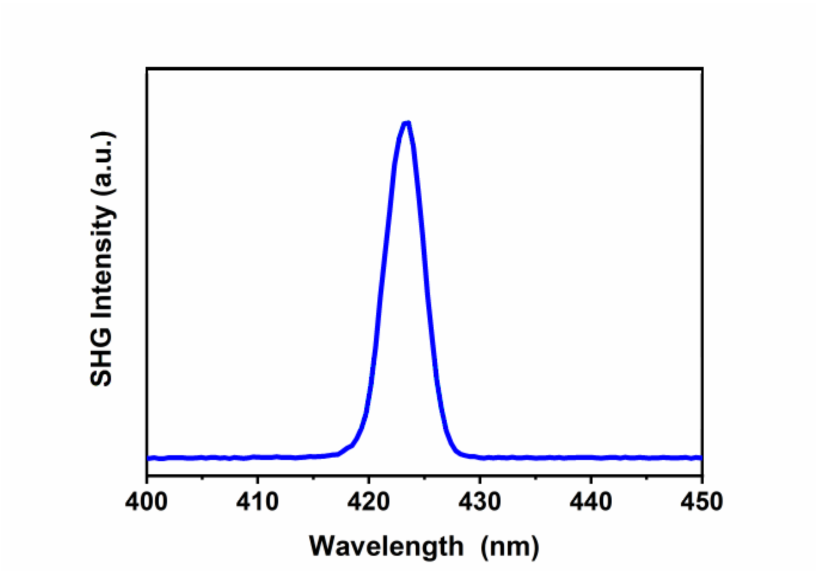

Figure S4. Second harmonic generation signal under 850 nm laser proves that TSFC belongs to non-centrosymmetric space group.

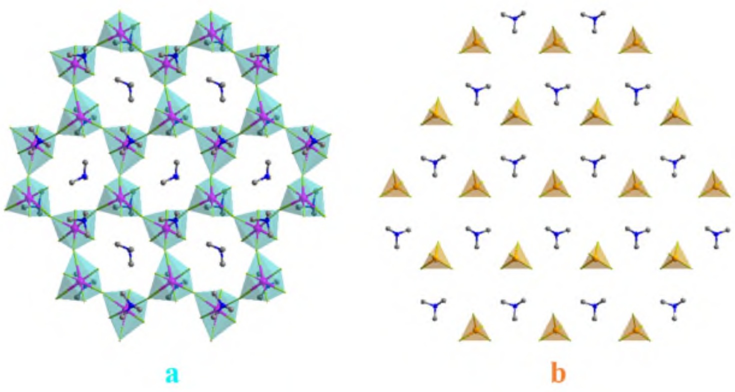

Figure S5. The projection along *c*-axis of a layer constructed by corner sharing $SbCl_6$ octahedra (left) and b layer formed by $FeCl_4$ tetrahedra for intercalation perovskite TSFC (right).

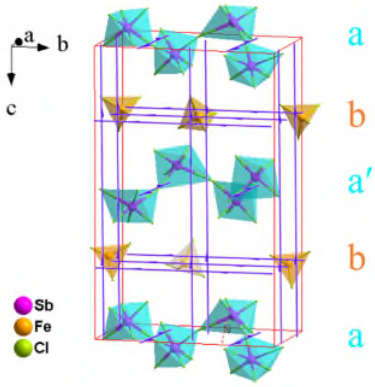

Figure S6. Symmetry elements of TSFC are shown in unit cell. Blue lines represent the 2_1 screw axis.

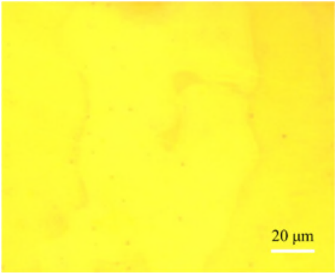

Figure S7. The optical microscope image without polarized light of TSFC.

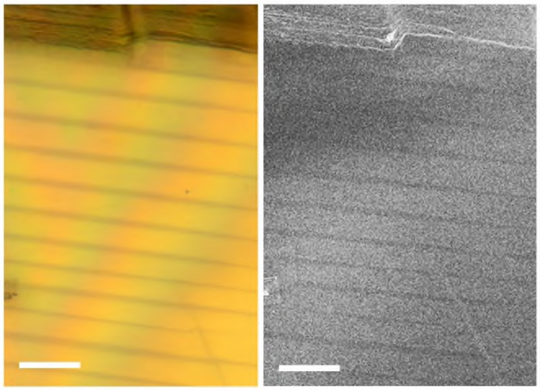

Figure S8. (a) The domain patterns in polarized light microscope (left) are consistent with top-view SEM image (right). Scale bar 10 um.

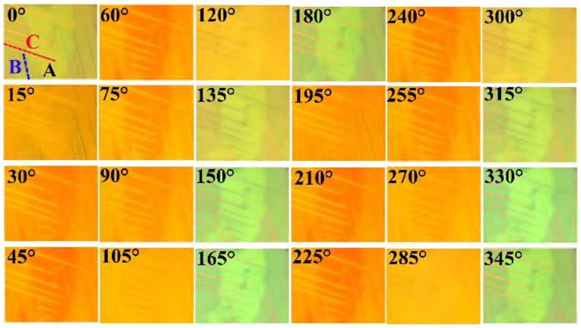

Figure S9. Polarized microscope images of the TSFC crystal along different angles. Under the polarized light microscope, although some contrast comes from surface uneven topography, it is significantly weaker than the contrast due to ferroelastic domains. The big contrast at different polarized angles originate mainly from the different ferroelastic domain. Form the figure, we can know the A domains have the biggest contrast at 60° and 150°, and the B domain have the biggest contrast at 0° and 90°. For domain C, the biggest contrast can be observed at 120° and 210°.

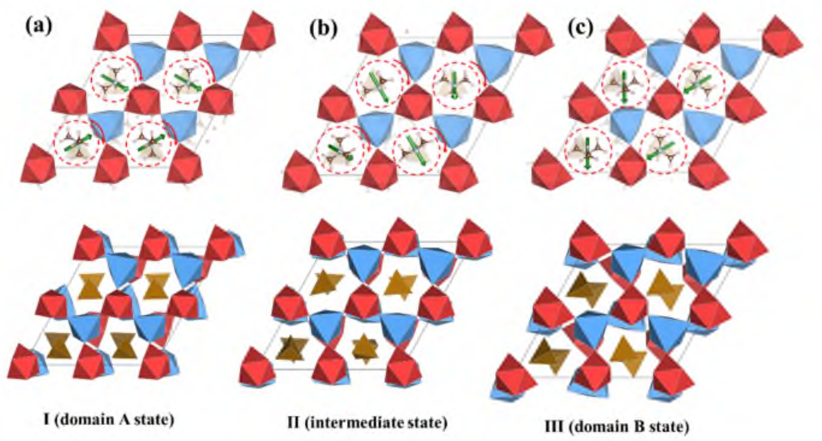

Figure S10. Motions of TMA and $FeCl_4$ during the ferroelastic transition (a) in I (domain A state), (b) II (intermediate state), and (c) III (domain B state)

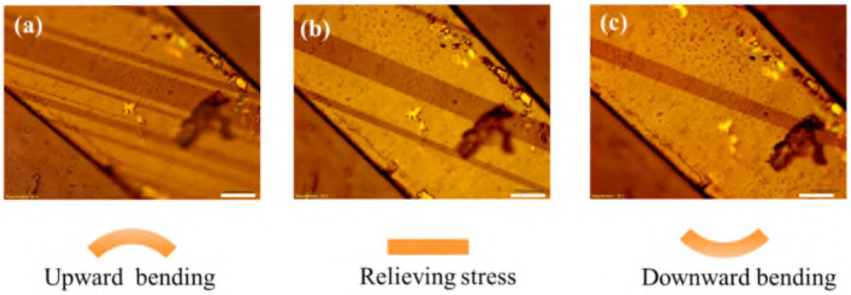

Figure S11. Changed ferroelectric domains structure of TSFC (a) under upward bending, (b) unbend and (c) downward bending sequence can be observed by polarized light images. Scale bar 50 μm.

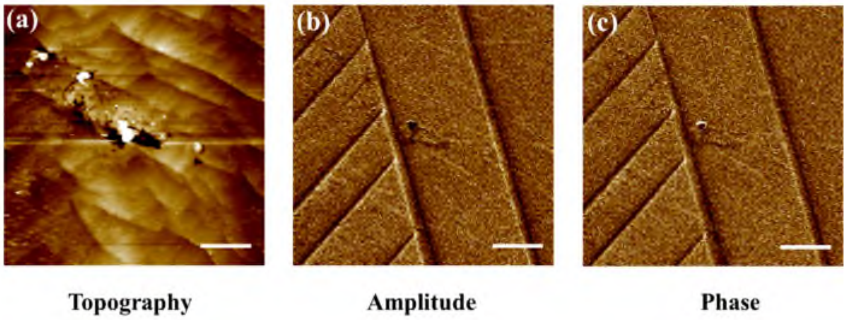

Figure 12. PFM topography (a), amplitude (b), and phase (c) images for as-growth intercalation perovskite TSFC crystal. Scale bar 10 μm.

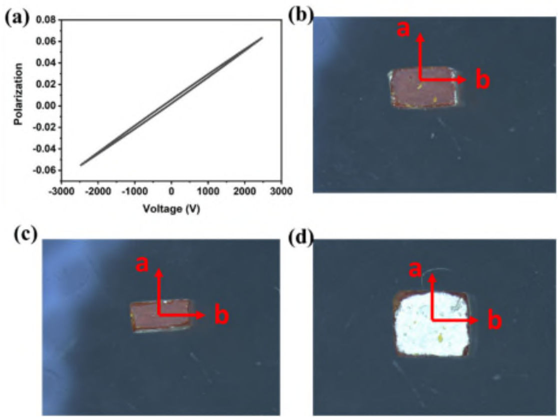

Figure 13. (a) Polarization vs electric field curve and (b, c, d) the diced single crystals were covered with silver paste along *b*, *a* and *c*-axis for TSFC.

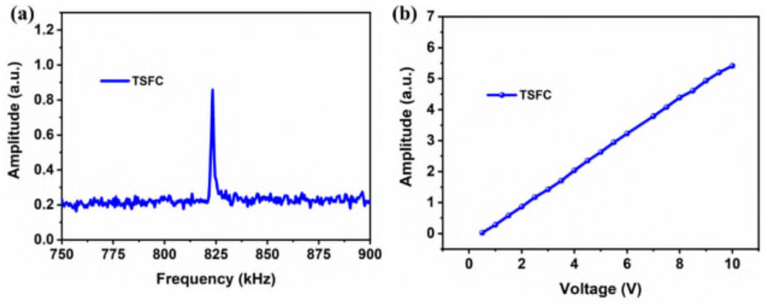

Figure 14. Piezoelectric properties of TSFC. (a) PFM resonance peaks at AC 2 V and (b) shear piezoelectric performance of intercalated perovskite TSFC.

Table S1 Crystal data and structure refinement for $(TMA)_3Sb_2Cl_9$ and $(TMA)_4Sb_2Fe(iii)Cl_{13}$.

	$(TMA)_3Sb_2Cl_9$		$(TMA)_4Sb_2Fe(iii)Cl_{13}$
Empirical formula	$C_9N_3H_{30}Sb_2Cl_9$		$C_{12}N_4H_{40}Sb_2Fe(iii)Cl_{13}$
Formula weight	742.91	742.91	1000.68
Temperature (K)	293(2)	380(2)	293(2)
Crystal system	Monoclinic	Monoclinic	Orthorhombic
Space group	Pc	$P2_1/c$	$P2_12_12_1$
Cell parameters Å	a = 10.0882(3)	a = 10.1822(7)	a = 8.9990(2)
	b = 9.1006(3)	b = 9.0586(6)	b = 16.201(3)
	c = 15.5447(4)	c = 15.7971(11)	c = 25.826(5)
β	90.0490 (10)	90.996	90°
V (Å^3)	1427.14	1456.85	3765.4
Z, ρ_{cal}. (g/cm^3)	2, 1.729	2, 1.694	4, 1.765
F(000)	720	720	1956
Theta range (°)	3.015 to 29.616	3.010 to 29.564	2.679 to 30.55
Limiting indices	$-14 \leq h \leq 14$	$-14 \leq h \leq 14$	$-12 \leq h \leq 12$
	$-12 \leq k \leq 12$	$-12 \leq k \leq 12$	$-23 \leq k \leq 23$
	$-17 \leq l \leq 21$	$-21 \leq l \leq 21$	$-36 \leq l \leq 36$
Reflections collected / unique	28663 / 6953	35276 / 4069	84031 / 11531
Data/restraints/parameter	6953 / 140 / 251	4069 / 84 / 167	11531 / 18 / 301
Completeness	99.7 %	99.8 %	99.8 %
GOF	1.153	1.029	1.099
Final R indices [$I > 2\sigma(I)$]	R_1 = 0.0318	R_1 = 0.0538	R_1 = 0.0314
	wR_2 = 0.0686	wR_2 = 0.1299	wR_2 = 0.0889
R indices (all data)	R_1 = 0.0398	R_1 = 0.0817	R_1 = 0.0387
	wR_2 = 0.0732	wR_2 = 0.1486	wR_2 = 0.0988

Table S2 Fractional Atomic Coordinates (×10^4) and Equivalent Isotropic Displacement Parameters ($Å^2$×10^3) for $(TMA)_3Sb_2Cl_9$ and $(TMA)_4Sb_2Fe(iii)Cl_{13}$. U_{eq} is defined as 1/3 of the trace of the orthogonalised U_{IJ} tensor.

$(TMA)_3Sb_2Cl_9$					$(TMA)_4Sb_2Fe(iii)Cl_{13}$				
Atom	*x*	*y*	*z*	U(eq)	Atom	*x*	*y*	*z*	U(eq)
Sb1	6166.1(3)	2113.4(4)	3288.1(2)	40.38(12)	Sb1	1583.4(3)	6575.1(2)	9433.6(2)	38.73(8)
Sb2	3163.6(3)	2881.4(4)	6565.3(2)	39.28(12)	Sb2	2184.7(3)	3365.4(2)	10588.8(2)	40.75(8)
Cl1	7874(2)	1765(3)	2167.4(14)	63.6(5)	Cl2	4112(2)	4109.6(10)	11033.3(7)	63.2(4)
Cl2	7348(2)	393(2)	4172.3(14)	60.7(5)	Cl3	3658(2)	5927.2(12)	8964.7(7)	66.5(4)
Cl3	7444(2)	4120(2)	3926.2(16)	67.1(5)	Cl5	-134(2)	5820.3(11)	8905.8(7)	70.4(5)
Cl4	4648(3)	-507(3)	2721.1(18)	81.5(7)	Cl8	2600(2)	2164.9(9)	11095.3(7)	60.8(4)
Cl5	1410(2)	1117(2)	6140.5(14)	61.9(5)	Cl12	1595(2)	7675.7(10)	8737.7(7)	66.7(4)
Cl6	2004(2)	4836(2)	5836.6(14)	56.7(5)	Cl13	393(2)	3855.6(12)	11222.4(6)	66.9(4)
Cl7	1932(2)	3383(2)	7855.5(13)	57.0(4)	Cl1	1469(3)	5240.8(12)	10082.5(6)	73.2(5)
Cl8	4991(2)	5125(2)	7098.8(16)	65.6(5)	Cl6	4540(2)	2693.6(12)	9886.8(7)	67.0(4)
Cl9	4316(2)	2252(3)	4692.0(15)	71.4(5)	Cl4	-1108(2)	7305.8(13)	9852.0(6)	69.7(4)
N1	7639(6)	3258(7)	6857(5)	59.0(16)	Fe1	2458.3(10)	373.1(6)	12420.8(4)	53.4(2)
C1	7287(12)	2154(12)	6198(7)	91(3)	Cl7	1699(2)	47.0(15)	11652.1(8)	81.7(5)
C2	8177(12)	2519(11)	7622(6)	84(3)	Cl9	4531(2)	1081.8(15)	12372.7(11)	92.4(7)
C3	8576(11)	4328(12)	6516(9)	99(4)	Cl10	661(3)	1072.7(18)	12782.6(12)	106.9(9)
N2	1706(7)	8066(7)	8482(6)	63.3(18)	Cl11	2815(4)	-769(2)	12848.9(14)	133.1(13)
C4	1989(12)	7618(11)	7581(8)	90(3)	N1	-2449(6)	7934(3)	8756(2)	56.3(12)
C5	859(13)	6970(10)	8906(7)	91(3)	N2	2732(7)	3116(3)	8879(2)	59.5(13)
C6	1109(11)	9542(10)	8518(8)	84(3)	N3	2594(9)	6319(4)	7739(3)	78.9(18)
N3	4678(12)	8138(13)	4810(7)	78(2)	C4	-2045(11)	8820(5)	8697(4)	81(2)
C7	5067(19)	6738(18)	4485(11)	97(3)	N5	-2421(7)	5264(4)	10113(3)	68.4(15)
C8	5276(18)	8489(19)	5659(9)	98(3)	C6	-3982(11)	7872(8)	8956(4)	106(4)
C9	3229(13)	8245(15)	4913(9)	81(3)	C7	-2261(11)	7498(6)	8253(4)	92(3)
N3X	4660(30)	7080(30)	5250(20)	86(4)	C8	1735(19)	3805(6)	9036(4)	126(5)
C7X	4240(50)	6470(50)	4430(20)	92(5)	C9	1821(11)	2391(6)	8794(5)	100(3)
C8X	6140(30)	7230(40)	5230(30)	92(4)	C10	-2276(12)	5308(7)	10682(3)	96(3)
C9X	4090(40)	8390(40)	5610(20)	93(4)	C11	1527(18)	6705(10)	7422(5)	156(7)
					C12	-3761(17)	5624(8)	9926(6)	149(6)
					C13	3657(13)	3329(10)	8440(5)	164(7)
					C14	4066(13)	6613(9)	7627(5)	123(4)
					C15	-2277(16)	4409(7)	9955(5)	128(5)
					C17	2626(16)	5439(6)	7588(5)	136(6)
