# Supplementary material for: Intercalation-driven ferroelectric-to-ferroelastic conversion in a layered hybrid perovskite crystal
Source: Nat Commun. 2022 Jun 3;13:3104. doi: 10.1038/s41467-022-30822-6 (PMC9166815; doi:10.1038/s41467-022-30822-6)
Supplement: Supplementary file 3 — Lasing Reporting Summary [file 41467_2022_30822_MOESM3_ESM.pdf]

nature research

Corresponding author(s): LOH Kian Ping

Lasing Reporting Summary

Nature Research wishes to improve the reproducibility of the work that we publish. This form is intended for publication with all accepted papers reporting claims of lasing and provides structure for consistency and transparency in reporting. Some list items might not apply to an individual manuscript, but all fields must be completed for clarity.

For further information on Nature Research policies, including our data availability policy, see Authors & Referees.

Experimental design

Please check: are the following details reported in the manuscript?

1. Threshold

Plots of device output power versus pump power over a wide range of values indicating a clear threshold

☐ Yes
☒ No

Not applicable to our study since we are not using the laser to interact with the sample. The Laser source was used in this study to collect vibration data only. Moreover, our LSV device has a built-in power pack where the pump power cannot be changed.

2. Linewidth narrowing

Plots of spectral power density for the emission at pump powers below, around, and above the lasing threshold, indicating a clear linewidth narrowing at threshold

☐ Yes
☒ No

Not applicable to our study since we are not using the laser to interact with the sample. The Laser source was used in this study to collect vibration data only.

Resolution of the spectrometer used to make spectral measurements

☐ Yes
☒ No

Not applicable to our study since we are not using the laser to interact with the sample. The Laser source was used in this study to collect vibration data only.

3. Coherent emission

Measurements of the coherence and/or polarization of the emission

☐ Yes
☒ No

Not applicable. The outgoing light in our LSV device is cleaned up by a polarizer and then converted into circularly polarized light with a lambda/4 plate.

4. Beam spatial profile

Image and/or measurement of the spatial shape and profile of the emission, showing a well-defined beam above threshold

☐ Yes
☒ No

Not applicable. We used a HeNe laser generates which is a well defined beam.

5. Operating conditions

Description of the laser and pumping conditions
Continuous-wave, pulsed, temperature of operation

☐ Yes
☒ No

Not applicable to our study since we are not using the laser to interact with the sample. The Laser source was used in this study to collect vibration data only.

Threshold values provided as density values (e.g. W cm^{-2} or J cm^{-2}) taking into account the area of the device

☐ Yes
☒ No

Not applicable to our study since we are not using the laser to interact with the sample. The Laser source was used in this study to collect vibration data only.

6. Alternative explanations

Reasoning as to why alternative explanations have been ruled out as responsible for the emission characteristics
e.g. amplified spontaneous, directional scattering; modification of fluorescence spectrum by the cavity

☐ Yes
☒ No

Not applicable to our study since we are not using the laser to interact with the sample. The Laser source was used in this study to collect vibration data only.

7. Theoretical analysis

Theoretical analysis that ensures that the experimental values measured are realistic and reasonable
e.g. laser threshold, linewidth, cavity gain-loss, efficiency

☐ Yes
☒ No

Not applicable to our study since we are not using the laser to interact with the sample. The Laser source was used in this study to collect vibration data only.

8. Statistics

Number of devices fabricated and tested

☒ Yes
☐ No

The exact number of samples in each experiment is provided in the Materials and Method section.

Statistical analysis of the device performance and lifetime (time to failure)

☐ Yes
☒ No

Not applicable to our study since we are not using the laser to interact with the sample. The Laser source was used in this study to collect vibration data only.

March 2018
